# The clinical and economic benefits of capecitabine and tegafur with uracil in metastatic colorectal cancer

**DOI:** 10.1038/sj.bjc.6603215

**Published:** 2006-06-27

**Authors:** S E Ward, E Kaltenthaler, J Cowan, M Marples, B Orr, M T Seymour

**Affiliations:** 1 School of Health and Related Research, University of Sheffield, Regent Court, 30 Regent Street, Sheffield S1 4DA, UK; 2 Cancer Research Centre, Weston Park Hospital, Sheffield S10 2SJ, UK; 3 Weston Park Hospital, Sheffield S10 2SJ, UK; 4 Cancer Research UK Centre, University of Leeds, Cookridge Hospital, Leeds LS16 6QB, UK

**Keywords:** cost-effectiveness, chemotherapy, colorectal cancer, capecitabine, tegafur

## Abstract

Two oral fluoropyrimidine therapies have been introduced for metastatic colorectal cancer. One is a 5-fluorouracil pro-drug, capecitabine; the other is a combination of tegafur and uracil administered together with leucovorin. The purpose of this study was to compare the clinical effectiveness and cost-effectiveness of these oral therapies against standard intravenous 5-fluorouracil regimens. A systematic literature review was conducted to assess the clinical effectiveness of the therapies and costs were calculated from the UK National Health Service perspective for drug acquisition, drug administration, and the treatment of adverse events. A cost-minimisation analysis was used; this assumes that the treatments are of equal efficacy, although direct randomised controlled trial (RCT) comparisons of the oral therapies with infusional 5-fluorouracil schedules were not available. The cost-minimisation analysis showed that treatment costs for a 12-week course of capecitabine (£2132) and tegafur with uracil (£3385) were lower than costs for the intravenous Mayo regimen (£3593) and infusional regimens on the de Gramont (£6255) and Modified de Gramont (£3485) schedules over the same treatment period. Oral therapies result in lower costs to the health service than intravenous therapies. Further research is needed to determine the relative clinical effectiveness of oral therapies *vs* infusional regimens.

Colorectal cancer is the second most common cause of cancer death in the United Kingdom. The incidence rises with age, being rare in people under 40 years of age, and 40% of patients are over 70 years old (Rougier and Mitry, 2003). Approximately 20% of patients with colorectal cancer present with advanced disease (Young and Rea, 2000), that is, disease which is already beyond the scope of curative resection, and a further 20–30% go on to develop advanced disease at a later date. Thus, around 45% of patients diagnosed with this disease eventually die of it. The median survival of patients with inoperable colorectal cancer treated without anticancer therapy is around 6–9 months. The main aims of treatment for patients with inoperable colorectal cancer are to relieve symptoms, prolong survival, and improve quality of life.

In the UK, palliative chemotherapy is offered to an increasing number of patients with metastatic colorectal disease (Cunningham *et al*, 2002). Various regimens are used in the treatment of metastatic colorectal cancer. During the 1980s and 1990s, standard first-line chemotherapy was intravenous 5-FU with the biomodulator leucovorin (5-FU/LV), with practice in the USA favouring bolus administration schedules (Poon *et al*, 1989) while in Europe and the UK the less toxic but more complex protracted infusional schedules were common (De Gramont *et al*, 1997). After 2000, many countries moved over to routine first-line combination therapy, usually involving 5-FU/LV and irinotecan; however, this approach was not adopted in the UK, where the National Institute for Clinical Excellence (NICE) continued to recommend single-agent fluoropyrimidine therapy as the routine first-line treatment for most patients. The Mayo Clinic regimen (Poon *et al*, 1989) combines bolus intravenous 5-FU with the biomodulator leucovorin (5-FU/LV), and acts as a comparator in many international trials of new therapies. The main alternatives to the Mayo bolus regimen are infusional regimens such as the de Gramont regimen (de Gramont *et al*, 1997) and, in the UK, the ‘Modified de Gramont’ (MdG) regimen (Cheeseman *et al*, 2002). These infusional FU/LV regimens are associated with higher response rates, longer progression-free survival, and less acute toxicity than the Mayo schedule, but since they have not been shown to be clearly superior in terms of survival, the choice of regimen may depend on clinician and patient preference as well as available resources.

Oral chemotherapeutic agents may be taken at home and require fewer hospital visits. They may therefore offer advantages over the bolus and infusional intravenous regimens. Two oral treatments, capecitabine (Xeloda®, Roche) and UFT/LV (tegafur with uracil in combination with leucovorin, marketed as Uftoral®, Bristol-Myers Squibb), are licensed for use in the UK. 5-FU is unsuitable for oral use as it is largely destroyed by catabolism in the gastrointestinal tract before reaching general circulation. However, capecitabine is a 5-FU prodrug, activated to 5-FU itself by a three-step metabolic pathway in normal and tumour tissues. UFT also contains a 5-FU prodrug, tegafur, along with uracil, which is used to inhibit the degradation of 5-FU.

The aims of this study were to evaluate these two oral treatment strategies in comparison to standard intravenous chemotherapy regimens used in the UK and to determine whether oral drugs are cost-effective in the treatment of metastatic colorectal cancer.

## MATERIALS AND METHODS

### Evidence on effectiveness

A systematic review of the literature was performed to identify randomised controlled trials (RCTs) or meta-analyses that compared capecitabine or tegafur with uracil with 5-FU/LV regimens as first-line treatment for metastatic colorectal cancer. An extensive literature search was conducted in electronic databases (CancerLit, Cocharane Library, Medline, Embase, etc). ‘Population’ search terms (e.g. colorectal, colon, rectum, neoplasm, carcinoma, adenocarcinoma) were combined with ‘intervention’ terms (e.g. capecitabine, Xeloda, fluoropyrimidine, tegafur, Uftoral). Details of the literature searches were described previously (Ward *et al*, 2003). The reference lists of relevant articles were hand searched. Trials were assessed for the following outcome measures: survival rates, progression-free survival, tumour response, and time to treatment failure.

The relative benefits in terms of survival gain, progression-free survival gain, adverse effects, and quality of life were established for the oral drugs through the results of the phase III RCTs found in the literature search. Due to the paucity of data, only a narrative synthesis was undertaken.

### Comparator

Intravenous 5-FU/LV was chosen as the comparator for the economic analysis because at the time of the study it was the most common first-line treatment for metastatic colorectal cancer currently in use in the UK. Although the Mayo regimen was used as a comparator in international trials at that time, this was not universally accepted in the UK. Survey results suggested that the Mayo, de Gramont and MdG (Cheeseman *et al*, 2002) regimens were all widely used in the UK (Summerhayes, 2003). This is broadly supported by the results of an earlier survey (Seymour *et al*, 1997). We therefore chose to compare the oral drugs against the Mayo regimen and two infusional regimens: the de Gramont regimen and the MdG regimen.

### Resource utilisation data

Resources used in the administration of the oral chemotherapy and Mayo regimens were taken primarily from the trial protocol and were validated against published evidence, where available, and discussion with local clinicians. Resources used for the de Gramont and MdG regimens were taken from previously published studies (Iveson *et al*, 1999; Cheeseman *et al*, 2002).

Utilisation for each regimen was based on the recommended doses in patients of average size (1.75 m^2^) as follows: *capecitabine*, four tablets of 500 mg and one of 150 mg (total 2150 mg) twice daily for 14 days every 3 weeks; *UFT*, five capsules (500 mg tegafur and 1120 mg uracil) plus 90 mg oral LV, daily for 28 days every 5 weeks; *Mayo regimen*, 5-FU 750 mg plus *d,l-*LV 35 mg daily for 5 days every 4 weeks; *de Gramont regimen*, total doses over 48 h of 3500 mg 5-FU plus 700 mg *d,l-*LV every 2 weeks; *MdG regimen*, total doses over 48 h of 5600 mg 5-FU plus 350 mg *d,l-*LV every 2 weeks.

Patients undergoing oral therapies were assumed to attend one outpatient appointment each cycle. Patients on the Mayo regimen were assumed to attend five outpatient visits each cycle. The de Gramont regimen was developed as outpatient regimen, using ambulatory pumps; but despite this, for a combination of reasons including funding mechanisms and poorly developed ambulatory treatment services, it became common practice in the 1990s for UK units to admit patients to hospital for each treatment cycle. In contrast, the MdG regimen is now almost universally given in the outpatient setting. For the purposes of this analysis we have therefore included for historical interest the original de Gramont regimen, given on an inpatient basis, incurring two in-patient days per cycle. Patients on the MdG regimen, as used currently in the UK, are assumed to receive it as outpatients, incurring one outpatient attendance and two community nurse home visits to disconnect and maintain their infusion lines each cycle plus additional resource usage associated with line insertion and infusion pumps (Cheeseman *et al*, 2002).

Estimates of the costs of management of adverse events took into account hospitalisations, physician consultations and drug treatment costs (Ward *et al*, 2003). For the treatment of adverse events resource utilisation data for the oral drugs and the Mayo regimen were taken from published resource use studies (Ollendorf, 1999; Twelves *et al*, 2001) and unpublished data from trials (Roche, 2002), in consultation with UK clinicians. For the de Gramont and MdG regimens resource utilisation for adverse events was taken from a previous analysis of colorectal therapies (Lloyd-Jones *et al*, 2001). The cost of line complications for patients on the ambulatory MdG regimen was based on estimates of the frequency of occurrence and cost of treating complications. (James R, Mid Kent Oncology Centre, Maidstone, personal communication, 2002). Complications included re-siting of the line in 5% of cases. Resource usage estimates were combined with UK unit costs taken from Netten *et al* (2001). Given the uncertainty relating to estimation of adverse event costs, a sensitivity analysis was examined in which adverse events were excluded and only drug acquisition and administration costs were considered.

### Costs

Costs were calculated from the perspective of the National Health Service (NHS) in the UK. All costs were adjusted to the year 2002. Unit costs are reported in Table 1. No discounting has been applied given that the median overall survival of patients in the majority of studies in this analysis was around 12 months.

Drug acquisition costs were based on an individual with a body surface area of 1.75 m^2^, with allowance for wastage. VAT was calculated on all drug costs. Unit costs for drugs were based on the BNF (British National Formulary, 2002), which do not reflect any bulk purchase discounts that may be negotiated. Discussion with clinicians indicated that a significant discount is often obtained for leucovorin within the NHS, although the level of discount may vary between institutions and over time. The impact of this discount was considered in sensitivity analysis.

Costs of GP visits and District Nurse visits were taken from Netten *et al* (2001). Estimates for the cost of outpatient appointments were taken from the accounts of a local hospital as well as published sources (Netten *et al*, 2001). Given that the estimates of cost of outpatient appointments were subject to wide variation, a range of possible costs was tested in sensitivity analysis. One-off costs included education for patients on oral therapies, and line insertion and overnight admissions associated with the MdG regimen (Iveson *et al*, 1999).

### Length of treatment

A previous UK phase III trial showed that stopping chemotherapy after 3 months in stable or responding patients, then restarting upon progression, is noninferior to continuing chemotherapy in terms of survival (Maughan *et al*, 2003). On this basis, many UK clinicians offer treatment breaks from 12 weeks, and consequently treatment duration is highly variable. For the purposes of economic evaluation, it was assumed that all patients would be treated for 12 weeks. This assumption may, however, underestimate total treatment costs given that many patients continue beyond 12 weeks, and a proportion of patients who stop treatment at 12 weeks may resume treatment later. A sensitivity analysis was considered in which total treatment costs were based on median treatment times from the trials.

### Cost analysis

A cost-minimisation analysis was performed for comparisons of capecitabine and UFT/LV with the Mayo regimen, since the survival benefits were shown to be statistically equivalent. A cost-minimisation analysis was also performed for comparisons of capecitabine and UFT/LV with the infusional regimens. The infusional de Gramont and MdG regimens produce higher response rates and progression-free intervals than the Mayo regimen, but in the only direct comparative trial overall survival with the de Gramont regimen was not significantly increased compared with Mayo (de Gramont *et al*, 1997). In the absence of direct evidence of a survival advantage for infusional therapy, a cost-minimisation analysis was therefore considered appropriate.

### Sensitivity analysis

A number of assumptions were made in the base case analysis that could potentially influence the results of the cost effectiveness analysis. To understand the impact of these assumptions on the results of the analysis, they were tested in a sensitivity analysis. Firstly market research conducted by Bristol-Myers Squibb, supported by feedback from a number of different NHS trusts contacted by the authors, suggested that hospitals purchased leucovorin at a substantially discounted price. The impact of applying the reported average discount of 87% was tested in sensitivity analysis (Scenario A). Secondly, the estimation of drug costs was based on the mean dose intensity from the trials, rather than the doses specified in the Summaries of Product Characteristics (Scenario B). Thirdly, the impact of estimating total drug costs based on the median treatment times in the trials, rather than assuming a 12-week treatment period for all drugs, was considered (Scenario C). In addition, two scenarios were run to illustrate the impact of varying assumptions on the cost of outpatient appointments: in one scenario, outpatient appointments for both oral and infusional therapies were assumed to incur the same cost of £109 (Netten *et al*, 2001) (Scenario D1); in the other scenario, the outpatient appointments were costed, based on NHS reference costs, at £86 for an outpatient appointment for oral therapies (follow-up medical oncology appointment) and £212 for a daycase appointment for intravenous therapies (medical oncology day-case appointment) (Scenario D2). Finally, given the substantial uncertainty surrounding the estimation of adverse event costs, a scenario was examined in which adverse event costs were excluded from the calculation of total treatment costs (Scenario E).

## RESULTS

### Description of included studies

Three studies were identified which provided evidence on the use of capecitabine. This included two open label phase III RCTs (Hoff *et al*, 2001; Van Cutsem *et al*, 2001) and a study of pooled data from these two RCTs (Twelves, 2002). These studies compared treatment with capecitabine and the Mayo regimen. Two open label phase III RCTs of UFT/LV were identified (Carmichael *et al*, 2002; Douillard *et al*, 2002). The Douillard study compared UFT/LV with the Mayo regimen while the Carmichael study compared UFT/LV with a modification of the Mayo regimen.

### Assessment of effectiveness

Duration of response and overall survival were not found to be significantly different between capecitabine and 5-FU/LV. Overall response rates, assessed by the investigator, were significantly greater in both trials in the capecitabine group. This improved response rate with capecitabine was confirmed by an independent review committee in the Hoff trial (25.8 *vs* 11.6%, *P*=0.0001) and the pooled data (22.4 *vs* 13.2%, *P*<0.0001) (Table 2). There was no significant difference between the two groups in time to disease progression or death, and time to treatment failure (Table 2).

There were no significant differences in duration of response or survival between UFT/LV and 5-FU/LV in either trial (results not shown). Time to disease progression was slightly inferior for the UFT/LV group compared to the 5-FU/LV group in the Douillard study (3.5 *vs* 3.8 months, *P*=0.01; Table 2), although there was no difference in time to disease progression between UFT/LV and 5-FU/LV in the Carmichael study. Overall survival in the two groups was also the same (Table 2).

Treatment with capecitabine had an improved adverse effect profile in comparison with the Mayo regimen, with the exception of hand-foot syndrome and hyperbilirubinaemia. UFT/LV was associated with fewer adverse events than the 5-FU/LV regimen. Neither capecitabine nor UFT/LV was associated with an improvement in health-related quality of life.

### Indirect comparison of infusional 5-FU regimens and oral prodrugs

No direct trial evidence comparing oral drugs with infusional 5-FU regimens was identified. A comparison of the Mayo regimen against the infusional regimens was therefore undertaken. One RCT was identified comparing the Mayo regimen to the de Gramont regimen (de Gramont *et al*, 1997) along with other supporting evidence (Meta-analysis Group in Cancer, 1998; Cheeseman *et al*, 2002). The limited evidence base available suggested that the de Gramont regimen may be superior to the Mayo regimen in terms of progression-free survival and in relation to toxicity, but that there was no statistically significant survival benefit.

### Cost analysis

A cost minimisation analysis was undertaken. The estimated total treatment costs, based on a 12-week treatment period, are given in Table 3. Detailed breakdown of the costs of administration and adverse events are given in Tables 4, 5 and 6. A sensitivity analysis on total treatment costs using median treatment times in the trials was also undertaken (Table 7).

The total treatment costs of both capecitabine and UFT/LV were estimated to be lower than the treatment costs for the three intravenous regimens. The cost estimates for UFT/LV, the Mayo regimen and the MdG regimen were similar. The cost estimate for de Gramont regimen is substantially higher than for the MdG regimen, both in terms of drug costs and administration costs, demonstrating the cost savings achieved over recent years by adoption of MdG in the UK.

The sensitivity analysis showed that the cost estimates for capecitabine were robust to changes in the cost parameters (Table 7). Capecitabine offered cost savings relative to all three intravenous therapies under all scenarios. The cost savings offered by capecitabine were smallest in Scenario A in which an 87% discount was applied to the cost of leucovorin (Table 7). This discount reduced the cost of the intravenous regimens. In this scenario, the cost difference between capecitabine and the MdG regimen fell from £1353 in the basecase to £483. This scenario may reflect the actual cost of lecovorin for many NHS institutions, although the exact size of the discount received by individual institutions is not known. UFT/LV costs remained lower than costs for the intravenous regimens except in scenario D1 where outpatient appointments with and without chemotherapy were assumed to incur the same cost.

The impact of using median treatment times from the trials (Table 7, scenario C) is to increase the estimated cost savings from the oral chemotherapy drugs, except in the case of UFT/LV based on the Carmichael trial. In this trial, the median treatment time is 4 months for UFT/LV compared with 3.5 months for 5-FU and this produces a slight cost saving in favour of the Mayo and MdG regimens compared with UFT/LV.

## DISCUSSION

This study shows that the higher drug cost of the oral chemotherapy agents relative to conventional intravenous 5-FU therapies are offset by lower administration costs, resulting in an overall cost saving of oral therapies over intravenous therapies in the UK.

Two previous economic evaluations were identified comparing UFT/LV with 5-FU, based on the same South American study (Murad *et al*, 1997a, 1997b). Murad *et al* used a panel of six physicians from Brazil and Argentina to estimate the costs of UFT/LV *vs* an unspecified 5-FU regimen in each country, and found that UFT/LV resulted in a small (£198 and £753) cost saving, mainly in the area of adverse event management. More recently Maroun *et al* (2003) provided a cost comparison of UFT/LV against Mayo regimen 5-FU from a Canadian perspective, based on a retrospective study of the Canadian centres in the Carmichael and Douillard trials. Hospital admission and oncology outpatient clinic visits were the most significant cost drivers. The study found that patients on the oral regimen had fewer outpatient visits for administration, although this was partly offset by more days of hospitalisation (reasons for hospitalisation were not given). Overall, cost savings from UFT/LV (excluding drug costs) came to $Can3221 in 1996 values (£2013 in UK 2002 values) per patient over the entire course of treatment.

Our study showed that oral chemotherapy drugs offer advantages in relation to resource usage. Protracted infused regimens are resource-intensive not only in terms of nurses and doctors to administer the infusion but also in terms of pharmacy time and resources. Drugs need to be prepared by specially trained individuals in an isolated area. Although pharmacist time and disposable bags, tubing, etc have been included in our cost analysis, the use of oral drugs also offer the advantage of freeing up time and space in isolated areas and allowing specialised pharmacists to engage in other activities. It should be noted that the use of dose banding has not been assumed in our calculations and this will offer some savings for 5-FU chemotherapy. The saving is unlikely to influence the findings of the paper.

As oral therapies can be prescribed and monitored through outpatient appointments and outreach clinics, patients undergoing oral treatment may have less contact with medical staff than those undergoing intravenous therapies. While this has benefits of savings to the health service and less travel and time required from patients, there are also associated risks, especially of over-compliance in the face of serious side effects. Owing to this, patient selection and education are critical, and doctors and nurses require training to carry out these tasks. Not all patients will be suitable for oral therapies, as some may not be physically or mentally capable. A good relationship is required between patients, doctors and nurses to encourage patients to report their symptoms accurately.

Evidence suggests that patients prefer oral therapies to intravenous therapies, as long as efficacy is not compromised. This was shown specifically by a crossover arm in a UFT/LV trial (Borner *et al*, 2002), but has also been reported in other studies, and was repeated by patient representatives advising the NICE committee on guidance recommendations. It was suggested that patients' preference for oral therapies is based primarily on a reduction of the disruptive impact of chemotherapy on their lives and a greater feeling of control over management of their disease (NICE, 2003).

Our analysis showed that drug administration costs were sensitive to the cost of an outpatient appointment. Costs from different sources vary widely. A similar finding was reported in the Canadian study (Maroun *et al*, 2003), which also used a wide range of values in a sensitivity analysis. It would be useful for a detailed resource use study of chemotherapy appointments to be conducted to determine the precise cost of OP appointments for intravenous therapy compared with appointments for the prescription of oral drugs.

One small crossover trial has found that patients preferred UFT/LV treatment over treatment with 5-FU/LV(Borner *et al*, 2002). Perhaps surprisingly, the improved side effect profile of oral regimens when compared to the Mayo regimen, patient preference for oral regimens, and improved response rates (for capecitabine) did not translate into improved quality of life for patients on oral therapies. This may reflect insensitivity of assessment tools, poor compliance with assessments, or difficulties with timing of questionnaire. However, it may also sound a note of caution: perhaps the mild but chronic toxicities of oral therapy, although never reaching an NCI CTC grade to cause alarm, may impact as much or more on quality of life as the severe but short-lived toxicities of cyclical intravenous drugs.

The greatest uncertainty in this analysis results from the necessity to perform an indirect comparison of oral drugs with the infusional 5-FU regimens. Further research comparing the optimum infusional 5-FU regimens with oral drugs would allow these treatments to be compared directly, in terms of efficacy, toxicity and quality of life. Ongoing and planned projects are making these comparisons in the context of combination therapy including oxaliplatin, irinotecan or novel targeted agents. In addition, the ongoing MRC FOCUS2 trial, in frail and elderly patients, includes the first direct randomised comparison of MdG *vs* capecitabine, with or without oxaliplatin.

In this study, we have shown that the higher cost of the oral chemotherapy drugs for treatment of metastatic colorectal cancer is offset by the lower cost of administration compared with intravenous chemotherapy regimens. In the period since the studies used in this analysis were conducted, the treatment of colorectal cancer has moved on. In many countries, including the UK, combination cytotoxic chemotherapy is a common option, and attention is focussed on introduction of novel targeted drugs. However, with oral-FP-based combination schedules the choices of oral or intravenous fluoropyrimidines remain, and these issues may in time extend to oral analogues of irinotecan and oxaliplatin. The adoption of an oral chemotherapy regimen should of course not be based on cost alone, but on the careful demonstration of at least equivalent efficacy, with favourable toxicity and quality of life for patients; however, the careful balancing, as here, of drug costs and the associated costs of treatment should help doctors and health commissioners in their goals to offer choices to patients within the context of finite healthcare resources.

## Figures and Tables

**Table 1:** Unit costs (£2002)

**Item**	**Cost (£2002)**	**Source**
In-patient day	367	Netten *et al* (2001)
Outpatient day	111	Netten *et al* (2001)
Outpatient clinic appointment with chemotherapy	150	*Personal communication, Christie Hospital, Manchester, UK (2001)*
Outpatient clinic appointment without chemotherapy	80	Personal communication, Christie Hospital, Manchester, UK (2001)
Medical oncology outpatient follow-up	88	NHS Reference costs (2001)
Day-case appointment	223	NHS Reference costs (2001)
District nurse home visit	20	Netten *et al* (2001)
GP home visit	60	Netten *et al* (2001)
GP telephone consultation	23	Netten *et al* (2001)
Day-care visit	129	Netten *et al* (2001)
GP surgery consultation	19	Netten *et al* (2001)
GP clinic consultation	27	Netten *et al* (2001)
A and E visit	62	Netten *et al* (2001)
Other hospital visits	76	Netten *et al* (2001)
Line insertion	509	*Personal communication, Christie Hospital, Manchester, UK (2001)*
Line insertion	549	Personal communication, Christie Hospital, Manchester, UK (adjusted)
*Line insertion*	265	Iveson *et al* (1999)
Pump	66	Iveson *et al* (1999)
Consultant hour	88	Netten *et al* (2001)
District nurse hour	44	Netten *et al* (2001)
Staff nurse hour	28	Netten *et al* (2001)
5-FU 1000 mg vial	12.80	BNF no 43 (2002)
5-FU 5000 mg vial	64.00	BNF no 43 (2002)
5-FU 500 mg vial	6.40	BNF no 43 (2002)
5-FU 250 mg vial	3.20	BNF no 43 (2002)
LV 50 mg vial	19.41	BNF no 43 (2002)
LV 350 mg vial	90.98	BNF no 43 (2002)

**Table 2:** Summary of effectiveness results

**Capecitabine studies**							**UVT/LV studies**			
**Study**	**Hoff *et al* (2001)**		**Van Cutsem *et al* (2001)**		**Twelves (2002)**		**Douillard *et al* (2002)**		**Carmichael *et al* (2002)**	
Response rate-investigator assessed (%)	Capecitabine	5-FU/LV	Capecitabine	5-FU/LV	Capecitabine	5-FU/LV	UFT/LV	5-FU/LV	UFT/LV	5-FU/LV
	(*n*=302)	(*n*=303)	(*n*=301)	(*n*=301)	(*n*=603)	(*n*=604)	(*n*=409)	(*n*=407)	(*n*=190)	(*n*=190)
	Overall response, CR or PR		Overall response, CR or PR		Overall response, CR+PR		Total tumour response *n* (%)		Total tumour response *n* (%)	
	75 (24.8)	47 (15.5)*	26.6	17.9	25.7	16.7	48 (11.7)	59 (14.5)	20 (10.5)	17 (9)
			(*P*=0.013)		(*P*=0.0002)					
Response rate-independent review committee assessed	Overall response, CR or PR		Overall response, CR or PR		Overall response, CR+PR					
	78 (25.8)	35 (11.6)†	57 (18.9)	45 (15.0)	22.4	13.2				
					(p<0.0001)					
Median time to disease progression or death	Capecitabine 4.3 months (95% CI: 4.1–5.1)		Capecitabine 5.2 months		Capecitabine 4.6 months (95% CI: 4.3–5.3)		UFT/LV 3.5 months (95% CI: 3.0–4.4 months)		UFT/LV 3.4 months (95% CI: 2.6–3.8 months)	
	5-FU/LV 4.7 months (95% CI: 4.3–5.5) (*P*=0.72)		5-FU/LV 4.7 months (*P*=0.65)		5-FU/LV 4.7 months (95% CI: 4.3–5.4)		5-FU/LV 3.8 months (95% CI: 3.6–5.0) (*P*=0.01)		5-FU/LV 3.3 months (95% CI: 2.5–3.7) (*P*=0.591)	
	HR=1.03 (95% CI: 0.87–1.22)		HR=0.96 (95% CI: 0.81–1.14)							
	*Time to treatment failure*		*Time to treatment failure*		*Time to treatment failure*					
	Capecitabine: 4.1 months		Capecitabine 4.2 months		Capecitabine 4.2 months					
	5-FU/LV: 3.1 months		5-FU/LV 4.0 months		5-FU/LV 3.6 months					
	(*P*=0.19), Hazard ratio 0.90 (95% CI:0.76–1.06)		(*P*=0.89)		Median time to response 1.7 months for capecitabine and 2.4 months for 5-FU/LV					

PR=partial response, CR=complete response, HR=Hazard ratio.

* *P*=0.05,

† *P*=0.0001.

**Table 3:** Estimated total treatment costs (£2002)

	**Capecitabine**	**UFT/LV**	**Mayo**	**MdG (outpatient)**	**de Gramont (in-patient)**
Drug cost	464	892	189	394	563
Administration	113	64	839	650	1500
Adverse events	131	170	170	29	22
Total 28-day costs	708	1126	1126	1073	2085
One-off costs	7	7	0	265	0
					
Total treatment costs (based on 12 week period)	2132	3385	3593	3485	6255
Cost savings on capecitabine			−1461	−1353	−4123
Cost savings on UFT/LV			−209	−101	−2870

**Table 4:** Cost of Administration (£s 2002)

	**Capecitabine**	**UFT/LV**	**Mayo**	**MdG**	**de Gramont**
*Cyclical expenses*					
Cost of outpatient visits	80	80	750	150	
Pharmacy preparation			9	15	16
Nurse time			69	35	
Pump				65	
Community nurse visit				41	
Administration disposables			11	20	
Creatinine test	5				
IP visits					734
					
Total cyclical admin costs	85	80	839	325	750
Weeks in cycle	3	5	4	2	2
28-day admin costs	113	64	839	650	1500
					
One-off expenses					
Patient education Line insertion	7	7			
				265	

**Table 5:** Frequency of consultations and hospitalisations relating to adverse events over treatment period for capecitabine, UFT/LV and Mayo

	**Capecitabine**	**UFT/LV**	**Mayo**
*Consultations*			
Day care visits	0.88		0.72
GP surgery visits	0.85	0.51	0.60
GP telephone consultations	0.55		0.43
A and E		0 .27	
Clinic consultation		0.26	
Other hospital visit		0.36	
GP home visits	0.32		0.25
			
*Hospitalisations*			
Hospital days	1.24	3.16	1.60

*Sources*: Capecitabine and Mayo regimens taken from Twelves *et al* (2001) and Ollendorf (1999), UFT/LV taken from Roche (2002) and Pazdur *et al* (1999).

**Table 6:** Cost of adverse events (£s 2002)

	**Capecitabine**	**UFT**	**Mayo**	**MdG**	**de Gramount**
Consultations and hospitalisations	455	1162	586	139	139
Drug treatment of adverse events	41	17	153	33	33
Line complications				48	
Total cost	£656	£1240	£867	£220	£173
Cost per 28 day cycle	£131	£326	£170	£29	£22

*Sources*: For capecitabine and Mayo regimens taken from Twelves *et al*, for UFT/LV taken from Roche (2002) and Pazdur *et al* (1999). For MdG and de Gramount taken from Lloyd-Jones *et al* (2001) and Cheeseman *et al* (2002) except for line complications James R, Mid Kent Oncology Centre, Maidstone, personal communication, 2002).

**Table 7:** Results of sensitivity analysis (£2002)

				
**(A) Capecitabine**	**Capecitabine**	**Mayo**	**MdG**	**de Gramont**
Basecase				
Costs	£2132	£3593	£3485	£6255
*Cost difference (Capecitabine minus 5-FU)*		−£*1461*	−£*1353*	−£*4123*
*Sensitivity analysis*				
A: Price of leucovorin discounted by 87%	£2132	£3296	£2615	£4852
		−*£1164*	−*£483*	-*£2721*
B: Drug costs based on mean dose intensities prescribed in trials	£1867	£3536	£3485	£6255
		−£*1669*	−£*1618*	-£*4388*
C1: Treatment costs based on median treatment time from Hoff *et al* (Capecitabine – 4.3 months, 5-FU 4.6 months)	£3316	£5985	£5629	£10 419
		−£*2669*	−£*2313*	−£*7103*
C2: Treatment costs based on median treatment time from Van Cutsem *et al* (Capecitabine – 4.8 months, 5-FU 4.6 months)	£3700	£*5985*	£*5629*	£*10 419*
		−£*2285*	−£*1928*	−£*6718*
D1: OP appointments for oral chemotherpay and intravenou chemotherapy assumed to have equal cost	£2258	£3015	£3254	£6255
		−£*757*	−£*996*	−£*3997*
D2: OP appointments based on NHS reference costs	£2164	£4687	£3923	£6255
		−£*2523*	−£*1759*	−£*4091*
E: Adverse events costs excluded	£1738	£3084	£3400	£6188
		−£*1346*	−£*1662*	−£*4450*
				
				

**(B) UFT/LV**	**UFT/LV**	**Mayo**	**MdG**	**de Gramont**
Basecase				
Costs	£3385	£3593	£3485	£6255
*Cost difference (UFT/LV minus 5-FU)*		−£*209*	−£*101*	−£*2870*
Sensitivity analysis				
A: Price of leucovorin discounted by 87%	£2504	£3296	£2615	£4852
		−£*792*	−£*111*	−£*2349*
B: Drug costs based on mean dose intensities prescribed in trials	£3197	£3536	£3485	£6255
		−£*339*	−£*288*	−£*3058*
C1: Treatment costs based on median treatment time from Douillard *et al* (UFT/LV 3.8 months, 5-FU 3.8 months)	£4655	£4944	£4696	£8607
		−£*289*	−£*41*	−£*3952*
C2: Treatment costs based on median treatment time from Carmichael *et al* (UFT/LV 4 months, 5-FU 3.5 months)	£4899	£4554	£4346	£7927
		*£345*	*£553*	−£*3028*
D1: OP appointments for oral chemotherpay and intravenou chemotherapy assumed to have equal cost	£3460	£3015	£3254	£6255
		*£445*	*£206*	−£*2795*
D2: OP appointments based on NHS reference costs	£3404	£4687	£3923	£6255
		−£*1283*	−£*519*	−£*2851*
E: Adverse events costs excluded	£2875	£3084	£3400	£6188
		−£*209*	−£*524*	−£ *3312*
